# Genetic-Background Modulation of Core and Variable Autistic-Like Symptoms in *Fmr1* Knock-Out Mice

**DOI:** 10.1371/journal.pone.0017073

**Published:** 2011-02-22

**Authors:** Susanna Pietropaolo, Aurélie Guilleminot, Benoît Martin, Francesca R. D'Amato, Wim E. Crusio

**Affiliations:** 1 Institut de Neurosciences Cognitives et Intégratives d'Aquitaine, Université de Bordeaux and CNRS UMR 5287, Talence, France; 2 Laboratoire Traitement du Signal et de L'Image, INSERM U642, Rennes, France; 3 Université de Rennes 1, LTSI, Rennes, France; 4 Institute of Neurosciences, Italian National Research Council (CNR), via del Fosso di Fiorano 64/65, Rome, Italy; University of Akron, United States of America

## Abstract

**Background:**

No animal models of autism spectrum disorders (ASD) with good construct validity are currently available; using genetic models of pathologies characterized by ASD-like deficits, but with known causes, may be therefore a promising strategy. The *Fmr1*-KO mouse is an example of this approach, modeling Fragile X syndrome, a well-known genetic disorder presenting ASD symptoms. The *Fmr1*-KO is available on different genetic backgrounds (FVB *versus* C57BL/6), which may explain some of the conflicting results that have been obtained with these mutants up till now.

**Methods:**

*Fmr1* KO and their wild-type littermates on both the FVB and C57BL/6 genetic backgrounds were examined on a battery of tests modeling the clinical symptoms of ASD, including the triad of core symptoms (alterations in social interaction and communication, presence of repetitive behaviors), as well as the secondary symptoms (disturbances in sensori-motor reactivity and in circadian patterns of activity, epileptic events).

**Results:**

*Fmr1*-KO mice displayed autistic-like core symptoms of altered social interaction and occurrence of repetitive behaviors with additional hyperactivity. The genetic background modulated the effects of the *Fmr1* deletion and it appears that the C57BL/6 background may be more suitable for further research on core autistic-like symptoms.

**Conclusions:**

The *Fmr1*-mouse line does not recapitulate all of the main core and secondary ASD symptoms, but still can be useful to elucidate the neurobiological mechanisms underlying specific ASD-like endophenotypes.

## Introduction

Despite much recent research on autism spectrum disorders (ASD), no animal models with good construct validity are currently available [1]. To develop one, a promising strategy is the use of models of pathologies that are characterized by autistic features [2]. The *Fmr1* knock-out mouse (*Fmr1*-KO) is an example of this approach: it is a confirmed model of Fragile X syndrome (FXS), a genetic disorder due to a mutation in the *FMR1* gene leading to a lack of FMRP, a protein playing a pivotal role in synaptic functioning [3], [4]. As FXS patients often display autistic symptoms and approximately 30% of them meet the full diagnostic criteria for ASD [5], [6], [7], [8], [9], [10], these two syndromes may share some common underlying mechanisms [11]. *Fmr1*-KO mice present many characteristics of FXS, including macro-orchidism, hyperactivity, and cognitive deficits [12]. Although this model has been widely employed in the last years, the possible validity of the *Fmr1*-KO mouse as a model for ASD has not been demonstrated convincingly, for several reasons.

First, behavioral characterizations of *Fmr1*-KO mice have not yet systematically taken into account all clinical criteria used to diagnose ASD and, in addition, most studies on *Fmr1*-KO mice have focused on a limited number of behavioral tests only, which does not reflect the complexity of ASD symptomatology. The diagnosis of ASD is based on a triad of core symptoms, namely qualitative and quantitative alterations of social interactions, deficits in communication, and the occurrence of repetitive/perseverative behaviors [13]. Beside these main alterations, several secondary symptoms that are variable in occurrence and severity can be present. They include increased sensory reactivity [14], reduced prepulse inhibition (PPI) of the acoustic startle response ([15], [16], but see also [17], [18]), hyperactivity and sleep-pattern (circadian) alterations [19], [20], increased anxiety [21], and epileptic seizures [22]. Hence, a useful animal model should present behavioral features that resemble at least one of the ASD core symptoms. Of course, an ideal animal model for ASD would mimic all core autistic features (as assessed by multiple tests) in association with some of the secondary ones [23].

To our knowledge, the only core autistic-like symptoms that have been comprehensively tested in *Fmr1*-KO mice have been deficits in social interaction [24], [25], [26], [27], [28], [29]. The presence of repetitive/perseverative behaviors has been investigated only through the evaluation of deficits in putative measures of behavioral flexibility such as reversal learning and working memory [30], [31], [32], [33], [34], while to date no reports on possibly altered communication are available. In addition, only some of the secondary autistic-like characteristics, such as changes in emotionality [27], [34], [35] and activity [24], [27], [29], [32], [35], [36], [37] have been assessed. Acoustic startle and its PPI have been investigated [38], [39], [40], [41], [42], but not their dependency on acoustic stimulus intensity. Finally, neither abnormalities in circadian patterns of activity, nor the presence of spontaneous episodes of epilepsy (for evoked seizures see:[34], [38], [43], [44], [45], [46]) have been evaluated in *Fmr1*-KO mice.

The second problem is that studies of autistic-like deficits in the *Fmr1*-KO model present contradicting results. For example, *Fmr1*-KO mice have been reported to display enhanced [29], normal [25], [27], or reduced levels of social interest and interaction [24], [26], [28]. Similarly, they showed enhanced [38], [40], [41], [42], [47], unchanged [34], or reduced [39] PPI, accompanied by reduced [38], [40], [41], [42], [47] or unaltered [34], [39] startle reactivity. Apart from differences in experimental procedures, it is most likely that these disparate results are due to the use of different genetic backgrounds, most-frequently the FVB and C57BL/6 (B6). For example, previous studies have demonstrated that the *Fmr1* mutation had opposite effects on the sizes of the hippocampal intra- and infrapyramidal mossy fiber terminal fields, depending on the background [27], [48]. Surprisingly, apart from some of the secondary symptoms [34], [46], [49], a systematic study of the effects of the *Fmr1*-deletion on ASD-like features in these two backgrounds has not been conducted yet. The present study therefore investigated the possible validity of the *Fmr1*-KO mouse on both the FVB and B6 backgrounds as a model for ASD by assessing the occurrence of autistic-like alterations in behaviors relevant to the core ASD symptoms (deficits in social interest and recognition, alterations in social interaction and communication, occurrence of repetitive behaviors), as well as secondary symptoms (PPI deficits, presence of epileptic events, alterations in activity and its circadian patterns).

## Materials and Methods

### Animals

Subjects were adult (12±1 weeks old) male *Fmr1*-KO and their wild-type littermates. The original *Fmr1* knock-out mutation was generated using 129P2 stem cells [12]. Breeders of C57BL/6J-*Fmr1* ^tm1Cgr/^Nwu (B6) or FVB.129P2-*Fmr1* ^tm1Cgr^/J (FVB) were originally obtained from Neuromice.org (Northwestern University, IL 60208, USA; MGI ID: 1857169) and The Jackson Laboratory (Bar Harbor, ME 04609, USA; Stock number: 004624), respectively. Wild-type males were from either the C57BL/6J or the FVB.129P2-*Pde6b* ^+^ *Tyr^c-ch^*/AntJ strains, originally purchased respectively from Charles River (L'Arbresle, France) and The Jackson Laboratory (Bar Harbor, ME 04609, USA; Stock number: 004828). Breeding trios were formed by mating two heterozygous females with an appropriate wild-type male. After 2 weeks the sire was removed and the females were single caged and left undisturbed until weaning of the pups. Mice were weaned at 21 days of age and group-housed with their same-sex littermates (3–5/cage). On the same day, tail samples were collected for DNA extraction and subsequent PCR assessment of the genotypes as previously described [12].

Only litters including males of both genotypes (+/• and −/•) were used for experiments. A total of 61 subjects were subjected to behavioral testing: 31 on the B6 background (15 wild-type and 16 KO) and 30 on the FVB background (16 wild-type and 14 KO). A different batch of adult (10±1 weeks old) mice (5 behaviorally-naive animals per genotype/background) was employed for EEG measurements.

NMRI mice (30 males and 30 females) were used as stimulus animals in the social tests. This strain was chosen because it differs from both genetic backgrounds on which the *Fmr1* KOs are maintained. It is also commonly employed in studies of social behavior, because of its good levels of sociability [50], [51]. Juvenile (3 weeks old) males and adult (12 weeks old) virgin females of the NMRI strain were purchased from Janvier (Le Genest-Saint-Isle, France), housed in same-sex groups, and left undisturbed for a week before being used for testing.

All animals were housed in polycarbonate standard cages (33×15×14 cm in size; Tecniplast, Limonest, France), provided with sawdust bedding (SAFE, Augy, France) and a stainless steel wired lid. Food chow (SAFE, Augy, France) and water were provided *ad libitum*. The animals were maintained in a temperature- (22°C) and humidity- (55%) controlled vivarium, under a 12:12 hr light–dark cycle (lights on at 7 a.m.).

### Behavioral procedures

Behavioral tests commenced at 12±1 weeks of age, as follows. Starting on day 1, a three-compartment test for sociability and preference for social novelty was administered, followed on day 3 by a direct social interaction test with a juvenile male, and on day 5 by a spontaneous alternation test in a Y-maze. On day 7, prepulse inhibition of the acoustic startle reactivity (PPI) was tested followed on day 11 by circadian modulation of locomotor activity, and, finally, on day 13 by a direct social interaction test with an adult female. Tests that relied mainly on observations of spontaneous behavior were conducted first in order to minimize possible undesirable transfer effects; tests that involved stressful stimulation, such as the acoustic startle test, or required social isolation, such as the assessment of circadian activity and social interaction with a female, were conducted last. All behavioral tests were carried out during the light phase of the cycle.

All 61 subjects were tested in the three-compartment test, direct social interaction with a juvenile male, spontaneous alternation, and PPI. In the three-compartment test, data from one FVB-KO and one B6-WT mouse were lost due to a problem with video recording: the analyses were therefore conducted on 30 B6 (14 WT and 16 KO) and 29 FVB (16 WT and 13 KO). The circadian modulation of activity was tested on a subgroup of 57 mice (14 B6-WT, 16 B6-KO, 15 FVB-WT and 12 FVB-KO), while the test of direct social interaction with a female was conducted on a subgroup of the latter of 54 mice (12 B6-WT, 15 B6-KO, 15 FVB-WT and 12 FVB-KO).

All experimental procedures were in accordance with the European Communities Council Directive of 24 November 1986 (86/609/EEC) and local French legislation.

### Sociability and preference for social novelty in the three compartment test

#### Apparatus

**Figure 1:**
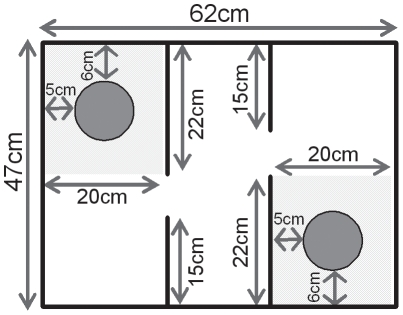
Schematic representation of the apparatus used for the three compartment test. The apparatus consisted of three rectangular compartments made of transparent Plexiglas. A stimulus cage (wire mesh, 10 cm in diameter, here represented as a dark grey circle) allowing visual, olfactory and partial tactile contact was placed in each side compartment. Performance in the task was evaluated across three 5-min trials, based on the relative time spent in the contact areas (highlighted in light grey).

The testing apparatus employed here is similar to the one previously described by others [52], [53]. It consisted of 3 compartments (Fig.1): a central chamber (45×18×25 cm) connected on each side to another compartment (45×20×25 cm) through a small rectangular opening (15×5 cm). The floors and walls (1 cm thick) of all compartments were made of transparent Plexiglas. Each side compartment contained a round stimulus cage (10 cm in diameter, 7 cm high) made of wire mesh (hole size: 0.7×0.7 cm) covered by a plastic roof (5 cm high). A metal weight was attached to the roof in order to keep the stimulus cage stable. Each stimulus cage was placed at a distance of 6 cm from the back wall and 4 cm from the sides. Tracking images from a camera above the center of the apparatus were analyzed with Ethovision (Version 3.1, Noldus Technology, Wageningen, The Netherlands).

#### Procedures

Experimental and stimulus mice (4-week old NMRI male mice) were individually housed in standard polycarbonate cages provided with sawdust, food, and water bottles and left undisturbed in the experimental room for about 10 min before testing began. Each experimental subject was then introduced in the middle of the central compartment and allowed to explore the apparatus for 3 trials of 5 min each:

- Trial 1 (habituation): the stimulus cages were empty; basal levels of exploration were assessed.
- Trial 2 (sociability): a stimulus mouse was introduced in one of the stimulus cages, while a novel object (a plastic grey cylinder, 6 cm in diameter, 2 cm high) was introduced in the opposite cage (sides were counterbalanced within experimental groups); preferential exploration of the social *versus* non-social novel stimuli was measured.
- Trial 3 (social novelty preference): as trial 2, but the object was exchanged for a novel stimulus mouse; preferential exploration of the novel *versus* familiar social stimulus was evaluated.

At the end of each trial the experimental animal was confined in the central compartment by means of two Plexiglas magnetic doors for 30 sec. At the end of the third trial the apparatus as well as the object and the stimulus cages were cleansed with water and dried.

#### Variables measured

Exploration of each stimulus was assessed by measuring the time spent in each contact area, a 20×22 cm area containing the stimulus cage (see Fig. 1). A percentage score was also computed for the last two trials as follows:

- On trial 2: Sociability score = 100×T_social stimulus_/(T_social stimulus_ + T_non-social stimulus_),
- On trial 3: Social novelty preference score = 100×T_novel social stimulus_/(T_novel social stimulus_ + T_familiar social stimulus_).

Finally, the total distance moved in the entire apparatus was measured in meters in each trial.

### Direct social interaction with a juvenile male

#### Apparatus

Direct social interaction was assessed in a 30×15×22 cm plastic cage with 3 cm of sawdust and a plastic roof with a 17×8 cm central opening.

#### Procedures

Experimental and stimulus mice were habituated to the experimental room as before. Each experimental mouse was then introduced into the testing cage and left to habituate for 5 min. An unfamiliar stimulus mouse (a 4-week old NMRI male) was then introduced into the testing cage through the roof opening. The testing session lasted 3 min, but was stopped immediately if aggressive episodes occurred. The testing cage was cleansed with water and the sawdust was renewed between sessions.

#### Variables measured

Testing sessions were recorded and videos were analyzed with Observer XT (version 7, Noldus, The Netherlands), taking only the experimental animal into account. One observer who was unaware of the genotype of the animals scored both frequency and duration for each of the following behavioral categories and elements [54], [55]:

- Affiliative behaviors: sniffing the head and the snout of the partner, its anogenital region, or any other part of the body; allogrooming (grooming the partner); traversing the partner's body by crawling over/under from one side to the other.
- Nonsocial activities: rearing (standing on the hind limbs sometimes with the forelimbs against the walls of the cage) and digging. Time spent in self-grooming (the animal licks and mouths its own fur) was analyzed separately, since this is sometimes considered representing repetitive behavior [23], [26], [54], [56], [57].

### Spontaneous alternation

#### Apparatus

Spontaneous alternation was assessed in a grey, plastic Y-maze, placed on a table 80 cm high and located in the middle of a room containing a variety of extramaze cues. The three arms of the Y-maze were similar in appearance and spaced at 120° from each other. Each arm was 42 cm long and 8 cm wide. The entire maze was enclosed by a wall 15 cm high and 0.5 cm thick. Tracking images from a camera above the maze were analyzed with Ethovision.

#### Procedures

Mice were habituated to the experimental room as before and then introduced at the end of one of the arms and allowed to explore the maze for 5 min. Allocation of the start arm was counterbalanced within experimental groups.

#### Variables measured

An entry into one of the arms was scored by an observer unaware of the genotype of the animals when all four paws of the animal were placed inside an arm. Spontaneous alternation, expressed as a percentage, refers to that proportion of arm choices differing from the previous two [58], [59]. Thus, if an animal made the following sequence of arm choices: A, B, C, B, A, B, C, A, the total number of alternation opportunities would be six (total entries minus two) and the percentage alternation would be 67% (four out of six).

### Prepulse inhibition of the acoustic startle reflex

#### Apparatus

The apparatus (SR-LAB, San Diego Instruments, San Diego, CA, USA) and procedures were previously described in detail [60], [61], [62], [63], [64]. Briefly, animals were acclimatized to the apparatus for 5 min. The first six trials consisted of six pulse-alone trials, two for each pulse intensity (100, 110, or 120 dB_A_), presented in a pseudorandom order. Subsequently, ten blocks of trials were presented. Each block consisted of three pulse-alone trials, one for each pulse intensity, three prepulse-alone trials (+6, +12, or +18 dB units above the background of 65 dB_A_), nine possible combinations of prepulse-plus-pulse trials (3 levels of pulse×3 levels of prepulse), and one no-stimulus trial (i.e., background alone). These 16 trials were presented in a pseudorandom order within each block, with a variable intertrial interval of a mean duration of 15 sec. The session was concluded with a final block of six consecutive pulse-alone trials as in the first block.

#### Variables measured

Reactivity scores obtained on the first and the last blocks of six consecutive pulse-alone trials were separately analyzed to measure startle habituation. The data obtained in the remaining trials were categorized into three main different subsets according to their relevance to distinct behavioral constructs [60], [61], [62], [63], [64]. First, startle reactivity was assessed by the reactivity scores obtained in the intermediate pulse-alone trials. Second, reactivity on prepulse-plus-pulse trials relative to middle pulse-alone trials was used to estimate prepulse inhibition. Third, to measure prepulse-elicited reactivity we included data from prepulse-alone and no-stimulus trials.

To better conform to the assumptions of parametric ANOVA, a natural logarithmic transformation was applied to the startle reactivity scores [60], [61], [62], [63], [64]. First, PPI was assessed by analyzing the raw reactivity scores on intermediate pulse-alone and on prepulse-plus-pulse trials. We analyzed the linear coefficients derived from each reactivity curve using a 3-way ANOVA with background and genotype as between- and pulse intensity as within-subject factors. Second, PPI was analyzed converting the reactivity data into percent scores (%PPI = 100× (pulse-alone − prepulse-plus-pulse)/pulse-alone) calculated for each subject for each of the nine possible prepulse-plus-pulse combinations and analyzed in a similar way as the raw scores. The analysis of both raw and percent scores is widely employed in mouse studies [60], [62], [65], [66], [67], [68] as a necessary precaution in case experimental groups show large differences in their startle reactivity.

### Circadian modulation of locomotor activity

#### Apparatus

The apparatus (Actimeter system, Imetronic, France) consisted of an isolated plastic cupboard (1.80 m high, 1 m wide, 0.6 m deep) containing 8 transparent plastic cages (21×11×17 cm) with a grid floor. A metal food dispenser and a water bottle were inserted in the front wall of each cage, while two horizontal lines of infrared captors (two for each line, interline distance = 25 mm, distance between two captors = 12.5 cm) were mounted along each of the longer side walls. The cages were illuminated 12 hrs per day starting from 7 a.m. The rack was connected to an electronic interface to communicate with a computer for automatic data storing.

#### Procedures

Each mouse was introduced into an activity cage at 6 p.m. and left undisturbed for the subsequent 25 hrs.

#### Variables measured

Locomotor activity was evaluated based on the number of breaks of the infrared captors. The first testing hour was analyzed separately in 10-min bins, in order to evaluate the locomotor response and habituation to a novel environment. The remaining 24 hrs were analyzed in 1 hr-blocks with the 12 hr-dark/light phase as a further within subject factor, in order to assess the circadian modulation of locomotor activity.

Immediately after testing, mice were housed singly in 30×15×14 polycarbonate cages (Tecniplast, Limonest, France) covered by a metal grid and with approximately 3 cm of sawdust on the floor.

### Direct social interaction with an adult female

#### Apparatus and procedures

Direct social interaction was assessed in the home cage in which the animals were isolated for about 36 hrs after the previous test. Experimental and stimulus mice were habituated to the experimental room as described for the previous experiments. An unfamiliar stimulus mouse (a 12-week old NMRI female) was then introduced into the testing cage and left there for 5 min.

During the test an ultrasonic microphone (Bat detector U30, Ultrasound Advice, UK) set on frequency division 10 was suspended 10 cm above the cage. Vocalizations were recorded using the Spectrogram 15 program (Visualisation Software LLC, sampling rate 48 kHz, format 16 bit) and analyzed with Avisoft SASLab Pro (Version 5. 013, Avisoft, Berlin, Germany) after a fast Fourier transformation (FFT). Spectrograms were generated with an FFT-length of 512 points, a time window overlap of 50% (100% Frame, FlatTop window), a frequency resolution of 488 Hz, and a time resolution of 1 ms. Call detection was provided by an automatic threshold-based algorithm and a hold time (0.04 s) mechanism.

#### Variables measured

Testing sessions were recorded and videos were analyzed with Observer XT, as described for the test with a juvenile male mouse. In addition, the frequency of mounting attempts was also recorded. Vocalizations were analyzed in terms of both frequency and mean duration.

### EEG analysis

#### Surgery

The occurrence of epileptic episodes was assessed following a classical protocol [69]. All animals were anesthetized (ketamine 60 mg/kg, xylazine 15 mg/kg, i.p.) and five monopolar tungsten rod electrodes were implanted and fixed to the skull with cyanoacrylate glue and acrylic cement. Four electrodes were placed bilaterally over the frontal and parietal cortex and one over the cerebellum (reference electrode). Animals were allowed to recover for at least two weeks before testing.

#### Apparatus and procedures

Mice were placed in a 15×15×30 cm Plexiglas cage containing sawdust within a Faraday cage. EEG recordings were collected during a 3 hr-session of free exploration which was repeated on 5 consecutive days. The electrodes were connected to the EEG apparatus with flexible wires and EEG activities were recorded using a digital acquisition system (Coherence 6.0.0.2, Deltamed-France, sampling rate 1024 Hz).

#### Variables measured

The presence of epileptic activity, absence seizures (characterized by spike-wave discharges) or interictal events was assessed by visual inspection of video-EEG recordings.

### Statistical analysis

All data were analyzed by ANOVA with genetic background B6 or FVB and *Fmr1* genotype (+/• or −/•) as between-subject factors. Within-subject factors were included as needed. Post-hoc comparisons were performed using Fisher's LSD test. Data are presented as mean ± SEM throughout.

All statistical analyses were carried out using SPSS® 13.0 for Windows (Release 13.0.1, SPSS Inc. Chicago IL, USA) and α was set at 0.05.

## Results

### Sociability and preference for social novelty in the three compartment test

#### Habituation (trial 1)

Animals did not show a preference for any compartment or contact area during the first 5 min-trial [all Fs<1, ns; data not shown]. No differences between backgrounds or genotypes were observed for locomotor activity [all Fs<1, ns; data not shown].

#### Sociability (trial 2)

**Figure 2:**
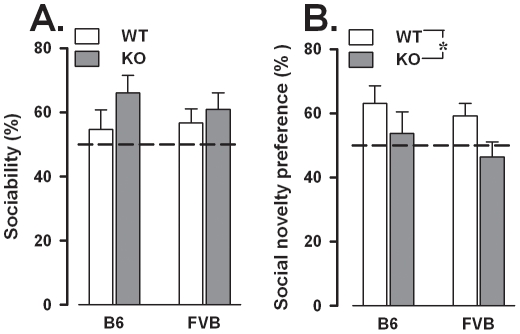
Sociability (A) and preference for social novelty (B) in the three compartment test. Exploration of each stimulus was assessed by measuring the time spent in each contact area, i.e., a 20×22 cm area containing the stimulus cage. Data are mean ± SEM. *p<0.05. The dotted line represents chance level (50%).

Mice preferentially explored the contact area containing the novel stimulus mouse compared to that with the novel object [F(1,55) = 24.13, p<0.0001]. This preference was similar in both genotypes [genotype × contact area: F(1,55) = 2.23, ns] and backgrounds [background × contact area: F<1, ns], as also confirmed by the analysis of the sociability scores [genotype: F(1,55) = 2.09, ns; background: F<1, ns; Fig.2A].

Locomotor activity did not differ between *Fmr1*-KO and WT mice, but was higher in the FVB compared to the B6 background [F(1,55) =  10.16, p<0.01; B6: 1.69±0.07, FVB: 2.19±0.14].

#### Social novelty preference (trial 3)

Mice preferentially explored the novel stimulus mouse compared to the familiar one [F(1,55) = 7.19, p<0.05]. However, this preference was absent in *Fmr1*-KO mice of both backgrounds [genotype × contact area: F(1,55) = 3.24, p = 0.08; contact area effect in WT: F(1,28) = 13.58, p<0.01, in KO: F<1, ns; social novelty preference scores, genotype: F(1,55) = 4.31, p<0.05; Fig.2B].

No differences in social recognition were observed between backgrounds [background × contact area: F<1, ns; social novelty preference scores, background: F(1,55) = 1.12, ns; Fig.2B]. *Fmr1*-KO and WT displayed comparable levels of locomotion but FVB were again more active than B6 [F(1,55) =  7.87, p<0.01; B6: 1.65±0.063, FVB: 2.09±0.15].

### Direct social interaction with a juvenile male

**Figure 3:**
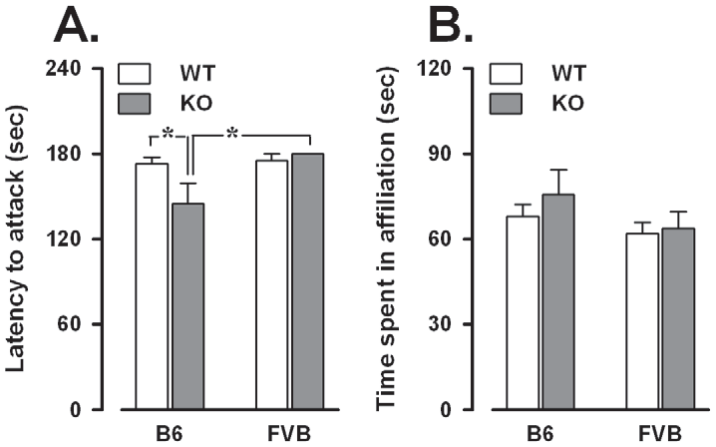
Aggression and affiliation in the direct social interaction with a juvenile male mouse. A: Attack latency (for non-attacking mice the maximum value of 180s was assigned). B: Time spent in affiliative behavior. Data are mean ± SEM. *p<0.05.

Surprisingly, some mice attacked the juvenile stimulus mouse before the end of the 3-min test causing early interruption of the encounter. These mice included 3 B6-WT (out of 15), 5 B6-KO (out of 16) and 1 FVB-WT (out of 16). In light of this unexpected finding we analyzed the latency to the first attack in all animals, assigning the maximum value of 180s to non-attacking mice (Fig.3A). The results showed that the *Fmr1* mutation caused an increased tendency to attack the juvenile stimulus, but only in the B6 background, B6-KO differing from both B6-WT and FVB-KO [background × genotype: F(1,57) = 3.99, p = 0.05, post hoc: p<0.05].

Subsequent behavioral analyses of the non-attacking animals demonstrated that *Fmr1*-KO did not differ from WT in the time spent performing affiliative behaviors (Fig.3B) or in their involvement in non-social activities, including self-grooming (data not shown). Furthermore, no background effects were detected for any behavior.

### Spontaneous alternation

*Fmr1*-KO and WT displayed comparable levels of spontaneous alternation (mean percent alternation rates varied between 50 and 64) and general exploration (mean numbers of entries varied between 41 and 51). In contrast, the two genetic backgrounds differed on both behavioral measures: FVB showed higher levels of spontaneous alternation [F(1,57) = 19.16, p<0.0001] and more entries into the arms of the maze [F(1,57) = 5.90, p<0.05] than B6.

### Acoustic startle response

One B6-KO exhibited a baseline startle value deviating more than 2SD from its group mean and was excluded from data analysis [61], [70]. Statistical analyses therefore used data from 30 B6 (15 WT and 15 KO) and 30 FVB (16 WT and 14 KO).

#### Acoustic startle habituation

**Figure 4:**
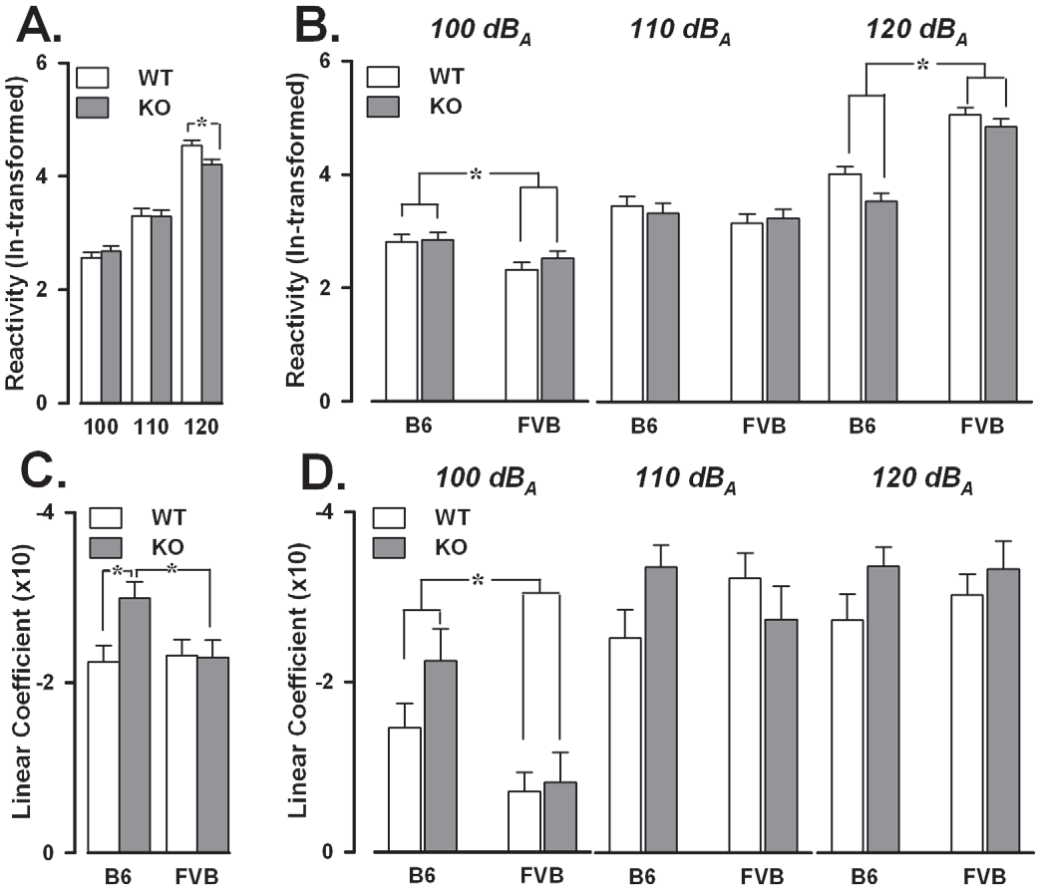
Startle reactivity and prepulse inhibition (PPI). A-B: Startle reactivity during the first and the last block of pulse alone trials; C–D: PPI expressed as the linear coefficient of the reactivity data. Data are mean ± SEM. *p<0.05.

There was a general reduction in the acoustic startle response from the first to the last block of pulse-alone trials [2-trial block effect: F(1,56) = 5.74, p<0.05], without any differences between genotypes or backgrounds (data not shown). Furthermore startle reactivity increased with pulse intensity [F(2,112) = 255.99, p<0.0001], an effect that was less prominent in *Fmr1*-KO of both backgrounds, showing a weaker startle response to the highest 120 dB_A_ pulse than WT [genotype × pulse: F(2,112) = 4.64, p<0.05, post hoc: p<0.05; Fig.4A]. Differences between the two backgrounds were detected in the overall levels of startle reactivity that were critically modulated by the intensity of the pulse stimulus: B6 showed stronger responses to the 100 dB_A_ and lower ones to the 120 dB_A_ pulses compared to FVB [background × pulse: F(2,112) = 61.1, p<0.0001, post hoc: p<0.05; Fig.4-B].

#### Pulse reactivity on intermediate trials

The analysis of the intermediate pulse alone trials confirmed the pattern of results observed during habituation. *Fmr1*-KO mice of both backgrounds showed lower levels of startle response to the highest 120 dB_A_ pulse level compared to the WT animals [genotype × pulse intensity: F(2,112) = 4.1, p<0.05, post hoc: p<0.05; ln-transformed reactivity to 120 dB_A_, *Fmr1*-KO: 4.64±0.09, WT: 4.36±0.08]. Background differences were found again: B6 showing stronger responses to the 100 dB_A_ and lower ones to the 120 dB_A_ pulses than FVB [background × pulse: F(2,112) = 66.35, p<0.0001, post hoc: p<0.05; ln-transformed reactivity, B6: 2.86±0.07, FVB: 2.40±0.07 at 100 dB_A_; B6: 3.99±0.08, FVB: 4.99±0.08 at 120 dB_A_].

#### PPI (Reactivity scores)

PPI was demonstrated by the negative values of the mean linear coefficients of the reactivity curves (Fig.4, C–D). The magnitude of PPI was enhanced in *Fmr1*-KO mice, but only on the B6 background [genotype × background: F(1,56) = 3.9, p = 0.05]. Post-hoc comparisons confirmed that B6-KO displayed higher levels of PPI than B6-WT and FVB-KO (Fig.4C). PPI was higher in B6 than FVB, but only at the lowest pulse level [Fig.4D; background × pulse [F(2,112) = 5.83, p<0.01].

#### Percent PPI

As expected [62], [65], [66], [71], PPI increased with prepulse intensity [F(2,112) = 11.04, p<0.0001] and the magnitude of this effect was modulated by the pulse level [pulse × prepulse: F(4,224) = 15.79, p<0.0001]. The analysis of the percent values led to a pattern of genotype and background differences similar to that described above (data not shown). The *Fmr1* mutation enhanced PPI, and this effect tended to be more prominent in B6, although the genotype × background interaction was not significant [F(1,56) = 2.89, p = 0.10]. Background differences in the expression of PPI were also confirmed, B6 showing higher PPI levels than FVB, but only at the lowest pulse intensity [background × pulse F(4,224) = 3.03, p = 0.05].

#### Prepulse reactivity

The reactivity on prepulse alone trials was also evaluated separately, including trials where only background noise was presented (data not shown). The startle response of all animals increased with the intensity of the prepulse stimulus [F(3,168) = 3.29, p<0.0001] and this effect was similar in both *Fmr1*-KO and WT. Animals from the B6 background showed higher levels of prepulse reactivity compared to FVB, but this difference was observed only at the highest 83 dB_A_ prepulse level [background × prepulse: F(3,168) = 3.29, p<0.05, post hoc: p<0.05; ln-transformed reactivity at 83 dB_A_, B6: 2.49±0.06, FVB: 2.33±0.06].

### Locomotor activity and its circadian modulation

#### Activity and locomotor habituation during the first testing hour

**Figure 5:**
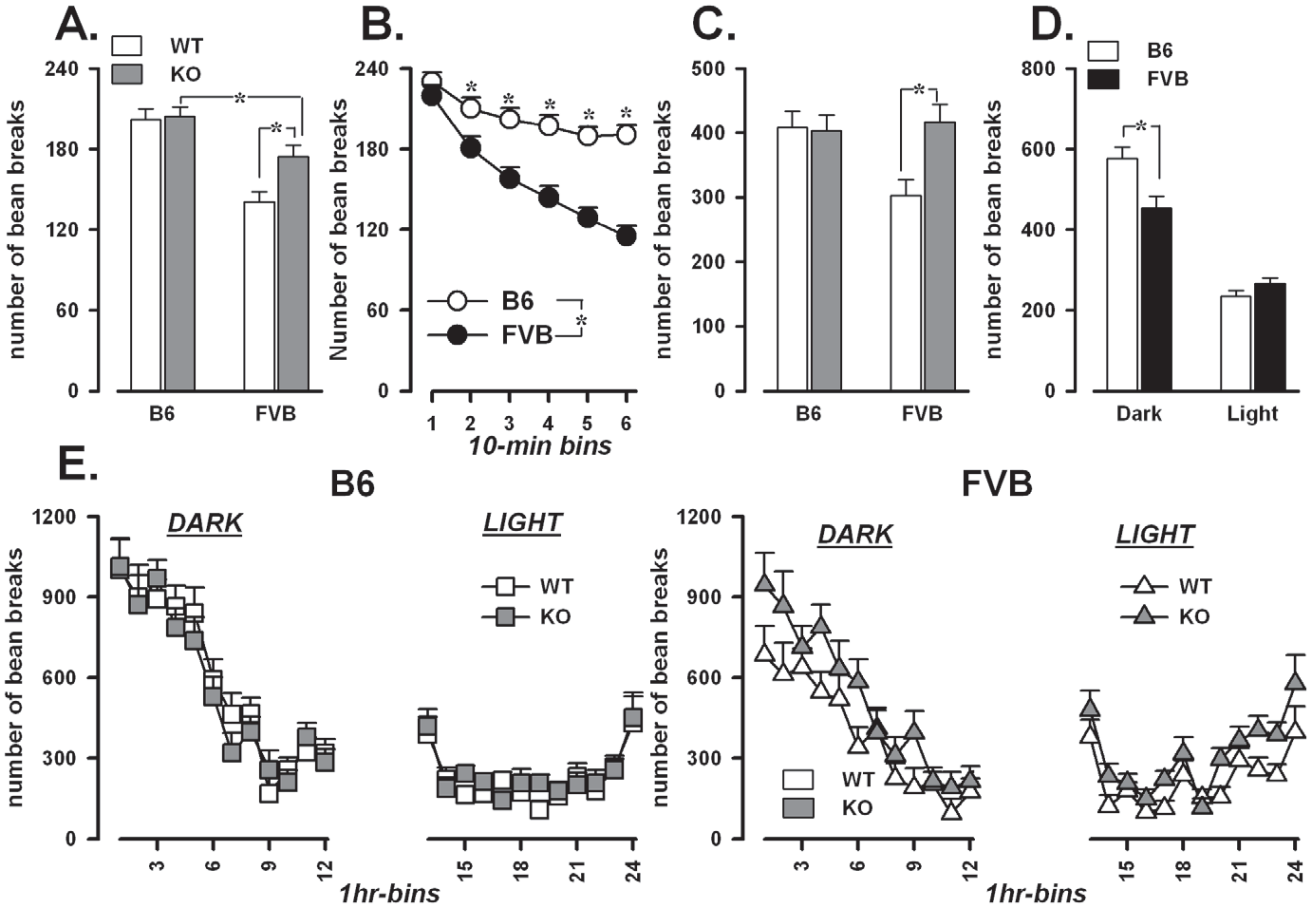
Locomotor habituation and circadian modulation of activity. A-B: Locomotion during the first testing hour; C–D: Total activity during the subsequent 24 hrs; E: 24 hr activity profile. Data are mean ± SEM. *p<0.05.

The first hour of testing was analyzed separately in 10-min time bins, in order to evaluate exploration of the novel environment and locomotor habituation (Fig.5A, B). Locomotor activity decreased over time [F(5,265) = 33.32, p<0.0001] and this was similar in *Fmr1*-KO and WT of both backgrounds (data not shown). Nonetheless, *Fmr1*-KO were overall more active during the first hour than their WT littermates [F(1,53) = 5.15, p<0.05] and this effect was larger in the FVB background [background × genotype: F(1,53) = 4.01, p = 0.05]. Post-hoc comparisons confirmed that FVB-KO were more active than both FVB-WT and B6-KO (p<0.05; Fig.5-A).

Differences between the two backgrounds were also observed (Fig.5-B, F(1,53) = 34.05, p<0.0001), FVB mice being less active and habituating faster [background × bins: F(5,265) = 6.40, p<0.0001] than B6.

#### Changes in locomotor activity during 24 hrs

The analysis of the subsequent 24 h (starting at 7 pm) confirmed the results observed during the habituation phase. *Fmr1*-KO were overall more active than WT [F(1,53) = 4.57, p<0.05], but this effect was mostly observed in the FVB background [background × genotype: F(1,53) = 5.43, p<0.05; post-hoc: p<0.05; Fig.5-C]. As before, B6 was more active than FVB, although only during the 12 hrs of darkness [background × light/dark phase: F(1,53) = 16.92, p<0.0001; post-hoc: p<0.05; Fig.5D]. The activity profile of *Fmr1*-KO of both backgrounds was comparable to that observed in WT (Fig. 5E, F). All animals showed highest levels of activity during the first 4-5 hrs of the dark period, which were drastically reduced during the light phase [1 hr-bin effect: F(11,583) = 4.007, p<0.0001; light/dark phase effect: F(1,53) = 200.39, p<0.0001, 1 hr-bin × light/dark phase: F(11,583) = 41.28, p<0.0001; Fig.5E].

### Direct social interaction with an adult female

**Figure 6:**
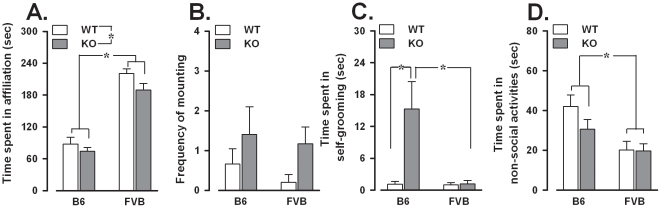
Social and non-social behaviors in the direct social interaction with an adult female. A: Time spent on affiliative behaviors towards an adult female. B: Frequency of attempts to mount the female. C: Self-grooming. D. Time spent on non-social activities. Data are mean ± SEM. *p<0.05.

*Fmr1*-KO spent less time in affiliative behaviors [F(1,50) = 4.78, p<0.05; Fig.6A] and showed a non-significant tendency to display more mounting attempts [F(1,50) = 3.08, p = 0.09; Fig.6B] compared to WT. It should be noted here that even if mounting attempts would have been classified as an affiliative behavior, the effect of the *Fmr1* mutation would have remained significant (data not shown). *Fmr1*-KO also showed higher levels of self-grooming, depending on the background: B6-KO spent more time performing self-grooming than both B6-WT and FVB-KO [background × genotype: F(1,50) = 5.07, p<0.05, post hoc: p<0.05; Fig.6-C]. Behavioral differences were also found between the two genetic backgrounds: FVB were more engaged in affiliative behaviors [F(1,50) = 149.62, p<0.0001, Fig.6-A] and less in non-social activities [F(1,50) = 5.69, p<0.05, Fig.6D] than B6.

#### Ultrasonic vocalizations

**Figure 7:**
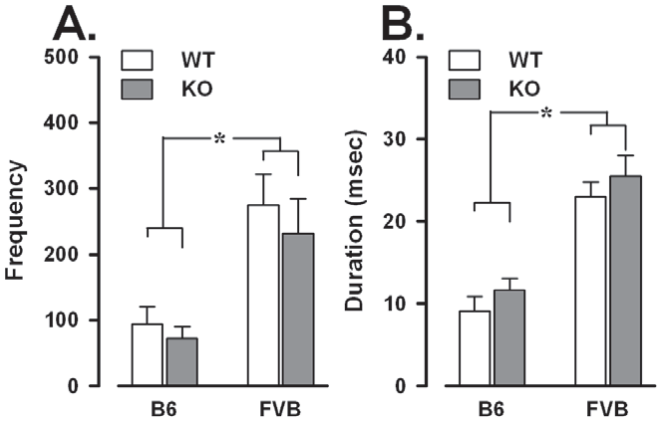
Ultrasonic vocalizations during the direct social interaction with an adult female. A: Frequency. B: Mean duration. Data are mean ± SEM. *p<0.05.

*Fmr1*-KO and WT emitted comparable ultrasonic vocalizations in terms of both frequency (Fig.7A) and duration (Fig.7B). However, there was a significant effect of background: FVB emitted a higher number of vocalizations [F(1,50) = 19.1, p<0.0001; Fig.7-A] and of longer duration [F(1,50) = 54.69, p<0.0001; Fig.7-B].

### EEG analysis

**Figure 8:**
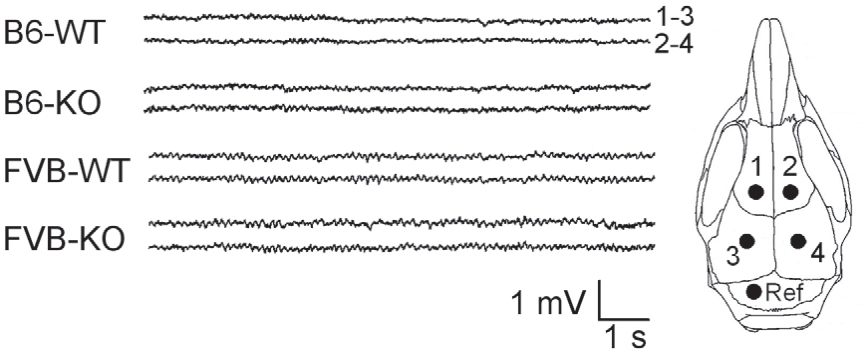
EEG analysis. Left: Examples of bipolar electrocorticographic recordings from each experimental group, illustrating the clear absence of any seizures, absences, or interictal events. Right: Electrode placements. 1 and 2: frontal cortex. 3 and 4: parietal cortex. Ref: cerebellar reference electrode.

*Fmr1*-KO of both backgrounds displayed EEGs which were comparable to those of WT (Fig. 8). No seizures, absences, or interictal events were observed in any animal during any of the five consecutive 3-hr recording sessions.

## Discussion

Our data show (i) that the *Fmr1* phenotype partially reproduces core symptoms of ASD with a very limited recapitulation of secondary autistic-like alterations and (ii) that the effects of the *Fmr1*-KO deletion are in part modulated by the genetic background. An overview of the results obtained is given in Table 1.

**Table 1:** Summary of the results.

Type of ASD-like symptom	Expected ASD-like symptoms	Behavioral test	*Fmr1*-KO Phenotype	Background of the *Fmr1*-KO phenotype	Differences between B6 and FVB backgrounds
***CORE***	***deficits in social interest***	*three compartment test (trial 2)*	normal levels of sociability	none	B6 moved less than FVB
***CORE***	***deficits in social recognition***	*three compartment test (trial 3)*	lack of preference for social novelty	B6 and FVB	B6 moved less than FVB
***CORE***	***aggressive tendencies***	*direct social interaction with a juvenile male*	shorter latency to attack the non-threatening social stimulus	B6 only	none
***CORE***	***deficits in social interaction***	*direct social interaction with a juvenile male*	normal levels of affiliation	none	none
***CORE***	***deficits in social interaction***	*direct social interaction with an adult female*	reduced affiliation	B6 and FVB	B6 showed less affiliation
***CORE***	***impaired communication***	*direct social interaction with an adult female*	unaltered ultrasonic vocalizations	none	B6 emitted fewer and shorter ultrasonic calls
***CORE***	***occurrence of repetitive behaviours***	*Y-maze*	normal levels of spontaneous alteration	none	B6 alternated and moved less
***CORE***	***occurrence of repetitive behaviours***	*direct social interaction with an adult female*	enhanced levels of self-grooming	B6 only	none
***SECONDARY***	***sensory hyper-response***	*acoustic startle test*	reduced acoustic startle response to strong stimuli	B6 and FVB	B6 showed lower startle response to the highest pulse level
***SECONDARY***	***PPI deficits***	*acoustic startle test*	increased PPI	B6 only	B6 had less PPI at the lowest pulse intensity
***SECONDARY***	***hyperactivity***	*first hr activity analysis*	overall hyperactivity	FVB only	B6 are more active
***SECONDARY***	***abnormal circadian activity patterns***	*24 hrs-activity analysis*	normal light-dark activity profile	none	B6 are more active in the dark phase
***SECONDARY***	***epileptic episodes***	*EEG recordings*	normal EEG, no signs of seizures	none	none

### Autistic-like symptoms in *Fmr1*-KO mice

*Fmr1*-KOs of both genotypes displayed a deficit in the preference for social novelty in the three compartment test, but had intact levels of sociability, in agreement with recent reports on the B6 [26] and the B6 × FVB backgrounds [25]. Despite the absence of ASD-like reduced levels of social interest, this lack of preference for social novelty may be interpreted as an autistic-like deficit in social recognition. Furthermore, *Fmr1*-KO of both backgrounds showed reduced social investigation, although this effect was dependent on the nature of the social stimulus, because it was observed only during the interaction with an adult female. This deficit does not seem to be due to reduced levels of sexual interest, since *Fmr1*-KO actually displayed more mounting attempts than WT (albeit non-significantly). Indeed, reductions in affiliative behaviors have been observed in *Fmr1*-KOs also during interaction with an ovariectomized female [28]. Besides these quantitative alterations, some qualitative changes were also observed in the social behavior of *Fmr1*-KOs in the B6 background. Here, an unexpected tendency to attack a juvenile stimulus mouse was found (i.e. a social stimulus that normally does not elicit aggressive responses). This resembles the signs of aggressiveness that have been reported in autistic patients [72], [73], [74], [75], [76]. In sum, it appears that *Fmr1*-KO display inappropriate responses in social situations.

B6-KO were also the only ones showing signs of repetitive behaviors by being more engaged in self-grooming during social interaction with an adult female. This result is corroborated by previous studies conducted in the B6 [26] and the B6 × FVB backgrounds [25], and it could be interpreted as a form of repetitive behavior due to increased emotional distress induced by the social context. Interestingly, increased self-grooming has been reported in other proposed mouse models for ASD as well (such as neuroligin-1 [56] and neurexin-1α KO mice [57], as well as the BTBR strain [54], [57]). Nonetheless, the occurrence of repetitive/perseverative behaviors seems to be modeled only partially by the *Fmr1*-KO, given our results in the Y-maze and previous findings from others on reversal learning in multiple backgrounds [34].

The B6 background was also more sensitive to the effects of the *Fmr1* mutation on PPI, but the direction of these effects was opposite to expectation: *Fmr1*-KOs displayed enhanced PPI. To our knowledge, there are no reports of increased PPI in ASD or FXS patients. On the contrary, most studies report PPI deficits in ASD [15], [16] (albeit subtle ones and with the exception of two experiments on younger subjects that reported no differences [17], [18]), and FXS patients display more severe PPI deficits [17], [40]. The magnitude of the acoustic startle response is also unaltered or even mildly increased in autistic and FXS patients [15], [16], [17], [40], in contrast to what is observed here in both backgrounds. Interestingly, these incongruent effects on PPI and startle have been consistently replicated across different mouse studies [38], [39], [40], [41], [42], [47]. Only one previous report has described a PPI deficit in B6-KO [39], but it did not use acoustic stimuli and measurements of whole body startle response. It should be noted that the enhanced PPI in B6-KO is not due to the decrease in startle response, as the effect on PPI is found at all pulse intensities, but for startle only at the highest pulse level. We therefore conclude that although sensorimotor response and gating are obviously influenced by *Fmr1*-KO in both humans and mice, the direction of the effects is opposite. The reasons for this discrepancy between the two species remain unknown; some authors have suggested a more important role of other FMRP-related proteins such as FXR2 in the mouse. This hypothesis needs further investigation, but is supported by the observation of PPI deficits in *Fxr2*-KO mice [77].

The only secondary autistic-like symptom displayed by *Fmr1*-KO mice was hyperactivity, although this was observed only in the FVB background and seems to be critically dependent on the testing conditions as the activity levels of *Fmr1*-KO were comparable to those of WT in all behavioral tests other than the 24 hr-monitoring. This result highlights the importance of test duration and apparatus, an issue that has been raised by previous studies (see [78] for review).

Further studies using more different test situations are needed to definitely exclude altered ultrasonic communication in the *Fmr1*-KO, for example by testing pup calls towards the dam [79], [80]. In addition, the absence of epileptic tendencies is surprising in view of the increased seizure susceptibility reported in both FXS and ASD. Our results, however, are in agreement with previous studies showing no difference in the responsiveness to chemical convulsants [38], but not with reports of increased sound- and kindling-induced seizures [34], [38], [43], [44], [45], [46].

### The impact of the genetic background

Our findings show that the genetic background partially modulates the *Fmr1* autistic-like phenotype, in such a way that certain alterations appear only in a specific background. As shown in Table 1, this modulation was rather subtle, since many important abnormalities were observed in both backgrounds. This pattern of results differs from the opposite effects found for the sizes of IIPMF terminal fields reported for the B6 and FVB backgrounds [27], [48]. It should be interesting to determine the genetic bases of this epistatic interaction, although that would be a far from trivial undertaking.

It should perhaps be noted here that “background” in this experiment not only consists of all genes in the genome differing between the FVB and B6 strains, but also by the pre- and postnatal maternal environment. There is no obvious way to control for this, barring laborious cross-fostering and embryo-transfer experiments. However, our observations of maternal behavior from PND 1 to 7 in heterozygous females did not detect any significant differences in the amount of maternal care (nursing postures, grooming of the pups) received by B6 and FVB pups, although B6 dams spent slightly more time in the nest (data not shown). While we cannot exclude that maternal effects are (partly) responsible for the background effects reported here, we feel that this is rather unlikely.

Based on our results, both backgrounds can be employed for modeling specific features of ASD. Even so, the B6 background seems to be the most suitable one for future autism research, since it presents more autistic-like core symptoms, in terms of both quantitative and qualitative alterations, even though this background already shows the lowest levels of social affiliation, ultrasonic vocalizations, and spontaneous alternation.

### Conclusion: Is the Fmr1-KO mouse a valid model for ASD?

The findings presented here clearly demonstrated that *Fmr1*-KO mice model only some specific autistic-like deficits. According to the view that the ideal ASD model should reproduce all core symptoms and some secondary ones [23], the *Fmr1*-KO should therefore be of limited validity only, especially in view of the lack of communication deficits that are reported here for the first time. However, some authors have suggested that the triad of core symptoms may, or even cannot, have a unitary explanation and should instead be fractionated and studied separately [81]. According to this view, a model displaying only some individual symptoms may still be valuable for future ASD research (as also suggested for other neuropsychiatric disorders [82]). Hence, the *Fmr1*-KO can still serve as a useful tool to investigate the neurobiology of specific ASD endophenotypes, with the design of novel therapeutic approaches as an ultimate goal.
